# Serotonergic Psychedelics in Neural Plasticity

**DOI:** 10.3389/fnmol.2021.748359

**Published:** 2021-10-12

**Authors:** Kacper Lukasiewicz, Jacob J. Baker, Yi Zuo, Ju Lu

**Affiliations:** Department of Molecular, Cell and Developmental Biology, University of California, Santa Cruz, Santa Cruz, CA, United States

**Keywords:** psychedelics, synapse, neural plasticity, 5-HT_2A_, psychoplastogen, psychiatric disorders

## Abstract

Psychedelics, compounds that can induce dramatic changes in conscious experience, have been used by humans for centuries. Recent studies have shown that certain psychedelics can induce neural plasticity by promoting neurite growth and synapse formation. In this review, we focus on the role of classical serotonergic psychedelics in neural plasticity and discuss its implication for their therapeutic potentials.

## Introduction

*To fathom Hell or soar angelic, just take a pinch of psychedelic*.

—Huxley and Osmond, 2018.

The term “psychedelic” was coined by the psychiatrist Humphry Osmond in 1956 in correspondence with the renowned author Aldous Huxley to remarket a class of compounds that induce profound changes in consciousness (Huxley and Osmond, 2018). Combining two Greek roots, *psyche* (soul, mind) and *deloun* (to manifest), the word means “mind-manifesting.” Previously, these compounds had been called “psychotomimetics” due to their ability to induce a transient, schizophrenia-like episode in healthy people (Gerard, 1956). Over the years, researchers have referred to this class of compounds variously as entheogens (“possessed by a god”) (Ruck et al., 1979), empathogens (“empathy-generating”) (Nichols et al., 1993), entactogens (“touching within”) (Nichols, 1986), and hallucinogens (Nichols, 2004), reflecting a constantly evolving perspective on their physiological and cultural impact.

The most recent term “psychoplastogen” emphasizes these drugs as catalysts for neuroplasticity in the brain (Olson, 2018). There is growing evidence that psychedelics promote the structural and functional plasticity of synapses (Ly et al., 2018; Inserra et al., 2021a; Kadriu et al., 2021), sites where neurons connect and communicate with each other. Interestingly, some structurally similar compounds share this feature but lack hallucinogenic effects (Cameron et al., 2021; Dong et al., 2021). As changes in synaptic circuits are widely believed to underlie learning and memory (Holtmaat and Svoboda, 2009), and numerous psychiatric disorders exhibit deficits in neuroplasticity (Goto et al., 2010; Forrest et al., 2018), psychedelics have attracted significant research interest in their molecular and cellular mechanisms of action (Inserra et al., 2021a; Kadriu et al., 2021), as well as in their therapeutic potential for psychiatric disorders such as addiction, depression, and anxiety (Carhart-Harris and Goodwin, 2017; Rucker et al., 2018; Dos Santos and Hallak, 2020).

Classical psychedelics fall into three main groups: tryptamines, ergolines, and phenethylamines (Nichols, 2016). They have diverse origins. For example, *N, N*-dimethyltryptamine (DMT) naturally occurs in mammalian brains (Saavedra and Axelrod, 1972); psilocybin is extracted from *Psilocybe* mushrooms (Hofmann et al., 1958) and mescaline from the peyote cactus *Lophophora williamsii* (Heffter, 1894); lysergic acid diethylamide (LSD) and 2,5-dimethoxy-4-iodoamphetamine (DOI) are semi-synthetic. They preferentially bind to the serotonin 2A (5-HT_2A_) receptor (Nichols, 2016), and the affinity is strongly correlated with the hallucinogenic effect (Glennon et al., 1984). Administration of ketanserin, a 5-HT_2A_ receptor antagonist, blocks the hallucinogenic effect of the classical psychedelic psilocybin (Vollenweider et al., 1998). Moreover, in 5-HT_2A_ knock-out (KO) mice, psychedelics do not produce the head-twitch response (high-frequency paroxysmal head rotation), a rodent proxy of hallucinatory behavior (Gonzalez-Maeso et al., 2007). A recent human study also reveals that the subjective intensity of psychedelic experience is correlated with 5-HT_2A_ receptor occupancy in the brain (Madsen et al., 2019). Therefore, signaling induced by 5-HT_2A_ receptor activation is likely necessary for psychedelics to cause hallucinations, even though many psychedelics bind to other receptors (*e.g*., 5-HT_1A_, other 5-HT_2_, and dopamine receptors) as well (Cumming et al., 2021). Since serotonin regulates mood, stress response, appetite, and reward processing (Berger et al., 2009), compounds with an affinity for 5-HT receptors may affect all these brain functions. As the pharmacology and downstream signaling pathways of psychedelics have been covered recently by excellent reviews (Lopez-Gimenez and Gonzalez-Maeso, 2018; Cumming et al., 2021; Kadriu et al., 2021), we will instead focus on the role of classical psychedelics in neural plasticity.

## Classical Psychedelics and Their Effects on Neuroplasticity and Neural Circuits

### *N, N*-Dimethyltryptamine (DMT) and Its Derivatives

DMT is an active ingredient in *ayahuasca* (“vine of the soul” in the Quechua language), a hallucinogenic drink consumed in shamanic rituals in South America. DMT can promote neuroplasticity both *in vitro* and *in vivo*. In cultured rat cortical neurons, DMT treatment for 24 h promotes the growth of neurites (axonal and dendritic processes emanating from the neuronal cell body), increasing their number, length, and complexity. The treatment also increases the density of dendritic spines (tiny protrusions from dendrites that are the postsynaptic sites of synapses). The increased spinogenesis is associated with synaptogenesis, as evidenced by an increase in the puncta density of VGLUT1, a presynaptic vesicular glutamate transporter. Likewise, a single high dose of DMT (10 mg/kg) increases synaptic density on pyramidal neurons in the prefrontal cortex (PFC) of adult rats. The structural changes are accompanied by functional changes, as shown by an increase in the frequency and amplitude of spontaneous excitatory postsynaptic currents (EPSCs) recorded *ex vivo* (Ly et al., 2018). In contrast, chronic, intermittent administration of a low dose of DMT (1 mg/kg) decreases PFC dendritic spine density in female, but not male, rats (Cameron et al., 2019). These findings suggest that DMT's psychoplastogenic effect is dose- and sex-dependent.

The molecular mechanisms underlying the neuroplastic effects of DMT are complicated. It likely involves 5-HT_2A_ signaling, as it can be blocked by co-treatment with ketanserin. Co-treatment with ANA-12, a selective antagonist of TrkB (tyrosine receptor kinase B), or rapamycin, an inhibitor of mTOR (mechanistic target of rapamycin), also blocks it, implicating TrkB and mTOR signaling in DMT-induced neuroplasticity as well (Ly et al., 2018). Furthermore, DMT is an agonist for the sigma-1 receptor, a chaperone protein at the endoplasmic reticulum that modulates calcium signaling (Fontanilla et al., 2009). As sigma-1 receptors can regulate voltage-gated ion channels and synaptic transmission (Kourrich et al., 2012), they may provide another path through which DMT exerts neuroplastic effects.

5-methoxy-*N, N*-dimethyltryptamine (5-MeO-DMT), a methoxylated derivative of DMT, is present endogenously in several species of plants, fungi, and the Sonoran Desert toad, *bufo alvarius* (Uthaug et al., 2019). It disrupts oscillatory neural activity in the PFC, the visual cortex (V1), and the mediodorsal nucleus of the thalamus (MD) in mice (Riga et al., 2014, 2018). Surprisingly, these effects are also observed in 5-HT_2A_ KO mice, but not in 5-HT_2A_ KO mice co-treated with WAY-100635, an antagonist of 5-HT_1A_ (Riga et al., 2018). It has been speculated that 5-MeO-DMT's action may depend on brain states: in anesthetized animals, it preferentially targets 5-HT_1A_ receptors on GABAergic inhibitory interneurons, whereas in awake animals it primarily binds to 5-HT_1A_ receptors on excitatory pyramidal neurons. Such state-dependent actions would differentially affect excitation/inhibition balance in the brain and may explain the opposite effect of 5-MeO-DMT on anesthetized vs. awake 5-HT_2A_ KO mice (Riga et al., 2018). Intracerebroventricular administration of 5-MeO-DMT increases the proliferation of neural progenitors and these newborn neurons possess more complex dendritic arbors and exhibit shorter afterhyperpolarization potentials as well as higher action potential thresholds compared to saline-treated controls (Lima da Cruz et al., 2018). Moreover, a mass spectrometry analysis of human cerebral organoids treated with 5-MeO-DMT reveals its modulatory effects on proteins involved in long-term potentiation, dendritic spine formation, microtubule dynamics, and cytoskeletal organization, highlighting the potential impact of 5-MeO-DMT on neuroplasticity (Dakic et al., 2017).

### Ibogaine and Its Analog

Ibogaine is a psychoactive β-carboline derivative isolated from the root bark of the West African rainforest shrub *Tabernanthe iboga* (Dybowski and Landrin, 1901). It has a high affinity for several targets including the *N*-methyl-*D*-aspartate (NMDA), the κ- and μ-opioid, and the 5-HT_2A_ receptors. Furthermore, ibogaine has the highest affinity for the sigma-2 receptor among psychedelics (Baumann et al., 2001; Ray, 2010). In humans, ibogaine is metabolized by cytochrome P450 2D6 (CYP2D6) into noribogaine (10-hydroxyibogamine, also referred to as 12-hydroxyibogamine in the Chemical Abstracts Service nomenclature) (Obach et al., 1998). Noribogaine shows higher plasma levels and remains detectable for a much longer period than ibogaine, suggesting that it may be the active compound *in vivo* (Mash et al., 2000; Baumann et al., 2001). Indeed, recent research has suggested that it may be noribogaine, rather than ibogaine *per se*, that stimulates neuroplasticity. In cultured rat cortical neurons, noribogaine increases dendritic arbor complexity while ibogaine does not. Co-treatment with ketanserin blocks such effect of noribogaine, implicating 5-HT_2A_ receptor activation (Ly et al., 2018). Unfortunately, a high dosage of ibogaine (≥ 50 mg/kg) can be neurotoxic: it induces cerebellar Purkinje cell degeneration in rats, possibly due to excitotoxicity from the inferior olive (O'Hearn and Molliver, 1993, 1997; Xu et al., 2000). The small safety margin between the therapeutic dose (40 mg/kg) and the neurotoxic dose raises concerns about ibogaine's safety for clinical use (Baumann et al., 2001). To address ibogaine's hallucinogenic and toxicity problems, researchers conducted function-oriented synthesis to develop tabernanthalog (TBG), a non-hallucinogenic, non-cardiotoxic analog of ibogaine (Cameron et al., 2021). TBG elevates the complexity of dendritic arbors of cultured rat cortical neurons and increases dendritic spine formation in the mouse somatosensory cortex *in vivo*. Such effects can be blocked by ketanserin co-treatment, indicating the involvement of 5-HT_2A_ receptor signaling. A more recent study (Lu et al., 2021) showed that a single dose of TBG can rescue the elevated anxiety, cognitive inflexibility, and sensory processing deficits in mice subjected to unpredictable mild stress (UMS). It also partially compensates for the UMS-induced dendritic spine loss, restores the electrophysiological properties of parvalbumin-expressing inhibitory interneurons, and normalizes baseline and sensory-evoked cortical neuronal activities.

### Psilocybin

Psilocybin is the active ingredient in so-called magic mushrooms (Geiger et al., 2018). In the body, psilocybin is quickly dephosphorylated into psilocin (Hasler et al., 1997), a compound that binds to several 5-HT receptor subtypes with relatively high affinity (Rickli et al., 2016). Both *in vitro* and *in vivo* studies on psilocybin have focused on its effect on excitatory synapses. They suggest that psilocybin promotes synaptic growth and strength. In cultured rat cortical neurons, psilocin increases dendritic arbor complexity and dendritic spine density (Ly et al., 2018). Likewise, a single dose of psilocybin promotes the formation of dendritic spines on layer 5 pyramidal neurons in the mouse medial frontal cortex *in vivo*, significantly increasing spine density; the psilocybin-induced new spines are no less stable than those formed under control conditions. In addition, psilocybin increases spine head size, which represents a strengthening of synapses (Shao et al., 2021). In the hippocampus and the PFC of pigs, a single dose of psilocybin also promotes synaptogenesis, as evidenced by a persistent increase in the presynaptic density of synaptic vesicle protein 2 (SV2A), a presynaptic integral glycoprotein that regulates neurotransmitter release (Raval et al., 2021). Another recent study (Hesselgrave et al., 2021) also shows that psilocybin increases synaptic strength, measured as an increased AMPA/NMDA ratio at the synapses between temporoammonic inputs and the distal dendrites of hippocampal CA1 pyramidal neurons in mice.

### LSD

LSD is an ergoline first synthesized by Albert Hofmann in 1938 from the lysergic acid found in ergot, a fungus that grows on grains (Hofmann, 1979). Despite the long-standing interest in LSD's cognitive effects, its cellular impact has only been explored recently. On the neural circuit level, LSD can modulate the spontaneous firing and burst-firing of reticular thalamus GABAergic neurons and disinhibit thalamocortical relay neurons in the mediodorsal thalamus in mice, as shown by *in vivo* electrophysiology (Inserra et al., 2021b). At the sub-cellular level, one study (Ly et al., 2018) on cultured rat cortical neurons found that 24 h LSD treatment significantly increases dendritic arbor complexity and dendritic spine density. There is a concurrent increase in synaptic density as measured by the co-localization of the presynaptic marker VGLUT1 and the postsynaptic marker PSD95. Although LSD binds to several 5-HT receptors with high affinity, its neuroplastic effects require 5-HT_2A_ receptor activation, as they are abrogated by co-treatment with ketanserin. Subsequently, it was shown (Ly et al., 2021) that shorter periods (15 min−6 h) of LSD stimulation already suffice to increase dendritic arbor complexity and spine density.

### DOI

Phenethylamines are probably the most extensively explored class of psychedelics due to their ease of synthesis (Nichols, 2018). Paradoxically, we know relatively little about the neuroplastic effects of most phenethylamines except DOI, which was first synthesized in 1973 (Coutts and Malicky, 1973). In cultured neurons, transient (1 h) DOI treatment increases dendritic spine size (Jones et al., 2009), and longer treatment increases the morphological dynamics of dendritic growth cones and neurite complexity, length, and number (Persico et al., 2006; Ohtani et al., 2014; Ly et al., 2018). In the mouse frontal cortex, DOI treatment selectively elevates the density of stubby and thin spines, but not of mature mushroom spines, on layer 2/3 pyramidal neurons; it also enhances long-term potentiation (LTP) at synapses onto such neurons (de la Fuente Revenga et al., 2021). *In vivo* studies further show that DOI promotes dendritic spine formation in the mouse sensory cortex without affecting spine elimination (Cameron et al., 2021).

DOI can also perturb cellular and network activity in the cortex. Extracellular recordings in the rat frontal cortex show that DOI reduces low-frequency oscillations and disrupts the temporal relationship between pyramidal neuron discharges and the local field potential (LFP). Both effects can be blocked by the 5-HT_2A_ antagonist M100907, implicating 5-HT_2A_ activation (Celada et al., 2008). In the primary visual cortex of awake mice, DOI reduces visually-evoked responses and surround suppression but spares basic retinotopic organization, tuning properties, and receptive field structure, which supports the idea that reduced bottom-up sensory drive underlies psychedelic-induced hallucinations (Michaiel et al., 2019).

The neuritogenesis- and synaptogenesis-promoting effects of DOI are blocked in culture when co-treated with ANA-12, ketanserin, or rapamycin, implicating signaling from TrkB, 5-HT_2A_, and mTOR, respectively, in DOI's downstream effects (Ly et al., 2018). Moreover, a single administration of DOI in 5-HT_2A_ knock-out mice does not increase the density of new synaptic spines in the frontal cortex, further suggesting that 5-HT_2A_ activation is critical for DOI induced neuroplasticity (de La Fuente Revenga et al., 2021). DOI's effects also depend on kalirin-7, a guanine nucleotide exchange factor that is a major regulator of dendritic spine morphogenesis in pyramidal neurons (Penzes and Jones, 2008), as incubating cultured cortical neurons in kalirin-7 interfering peptides, peptides that displace kalirin-7 from the postsynaptic density of dendritic spines, also blocks DOI's neuroplastic effects (Jones et al., 2009). However, there is evidence that a single administration of DOI in mice induces epigenetic changes in enhancer regions, regions of DNA that increase the likelihood that certain genes will be transcribed, that persist for at least a week after DOI administration, suggesting a molecular basis for long-term effects (de La Fuente Revenga et al., 2021).

### Ketamine

First synthesized in 1965 (McCarthy et al., 1965), ketamine is not a serotonergic psychedelic, but a non-competitive *N*-methyl-D-aspartate receptor (NMDAR) antagonist initially used as a general anesthetic. Notably, ketamine also produces significant psychological effects including hallucinations and dissociation from reality, not unlike the behavioral effects of psychedelics. Moreover, recent studies have highlighted its antidepressant effect (Nowacka and Borczyk, 2019). It is thus interesting to discuss ketamine's effects together with those of psychedelics.

Like classical psychedelics, ketamine also promotes neuroplasticity. Cultured rat cortical neurons significantly increase spinogenesis and synaptogenesis after 15 min of ketamine treatment (Ly et al., 2021). Similarly, transient ketamine treatment promotes the dendritic arborization of dopaminergic neurons either cultured from embryonic mouse mesencephalon or derived from human induced pluripotent stem cells (iPSCs) (Cavalleri et al., 2018). An enhancement in structural plasticity is also reported by multiple animal studies. For example, ketamine rapidly increases the spine density of L5 pyramidal neurons in the medial prefrontal cortex (mPFC) of rats (Li et al., 2010) as well as in hippocampal CA1 neurons in Flinders Sensitive Line (FSL) rats, a genetic model of depression (Treccani et al., 2019); it also significantly ameliorates the spine density decrease in the mPFC and hippocampus of mice subjected to chronic social defeat stress (Zhang et al., 2019). *In vivo* imaging studies further show that ketamine enhances spine formation in the mouse medial frontal cortex (Phoumthipphavong et al., 2016) and somatosensory cortex (Pryazhnikov et al., 2018), and partially restores spines lost during chronic exposure to corticosterone (Moda-Sava et al., 2019). Such plasticity is believed to underlie ketamine's rescuing effect on depression-like behaviors in rodent models (Moda-Sava et al., 2019). In addition to the structural plasticity, ketamine can elicit a form of functional synaptic plasticity reminiscent of homeostatic synaptic upscaling, providing another possible cellular mechanism for its antidepressant effect (Kavalali and Monteggia, 2020).

## Conclusion and Discussion

Since the early 20th century, there has been considerable interest in using psychedelics to treat mental illnesses (Carhart-Harris and Goodwin, 2017; Bogenschutz and Ross, 2018; Nichols and Walter, 2021). Numerous clinical studies have shown encouraging therapeutic potentials of psychedelics, particularly LSD and psilocybin, and to a lesser extent ayahuasca, ibogaine, and dipropyltryptamine (DPT), in treating disorders including addiction to alcohol, nicotine, or illicit drugs, anorexia nervosa, depression, distress and anxiety concerning death, obsessive-compulsive disorder, and post-traumatic stress disorder (Bogenschutz and Johnson, 2016; Johnson et al., 2019; Dos Santos and Hallak, 2020; Foldi et al., 2020; Krediet et al., 2020; Andersen et al., 2021; Dos Santos et al., 2021). The neuroplastic effects of psychedelics, however, have only been appreciated much more recently. Given that DMT, LSD, and DOI, representatives of three classes of psychedelics, as well as ketamine, all possess the ability to promote dendritic branching and dendritic spine formation, it is conceivable that the therapeutic effects of psychedelics are at least partially mediated through the reconfiguration of neuronal networks. The formation of new dendritic spines represents the addition of new synapses to the neuronal circuitry (Knott et al., 2006). The new synaptic connections will persist or be eliminated in an activity-dependent manner; their presence or disappearance will in turn shape the activity patterns of the neurons they reside on (Fu and Zuo, 2011). In addition, psychedelics may modulate dendritic excitability in a compartmentalized manner through interactions with 5-HT receptor subtypes that are co-expressed in the same neuron but with distinct subcellular localizations. The local control of membrane excitability will affect dendritic calcium signaling and, consequently, synaptic plasticity and alter synaptic integration (Savalia et al., 2021). Through such reciprocal structural and functional modifications, some new synapses may eventually outcompete the existing aberrant synapses, and the neuronal circuit may cease the abnormal firing that underlies mental illnesses. The physical change in neural circuit connectivity potentially explains the persistence of symptomatic improvements.

Psychedelics' molecular mechanisms of action are very complex (Nichols, 2016). While 5-HT_2A_R signaling is widely believed to be a major contributor to psychedelics' effects, some recent studies indicate that it is not the sole player. One study shows that ketanserin treatment does not abolish the antidepressant effect of psilocybin in mice (Hesselgrave et al., 2021); another suggests that psilocybin decreases 5-HT_2A_R density in the hippocampus and PFC of pigs while increasing synaptic density (Raval et al., 2021). Indeed, in addition to being agonists or partial agonists of the 5-HT_2A_R, all known psychedelics are also agonists of the 5-HT_2C_R. The 5-HT_1A_R is also likely involved, particularly for tryptamines and LSD (Nichols, 2004, 2016). Moreover, psychedelics may have an affinity for other receptors. For example, LSD is a high-affinity agonist of dopaminergic receptors (Watts et al., 1995). In fact, a screening of 35 psychedelics at 51 receptors, transporters, and ion channels suggests that the interaction profile of psychedelics is broader than generally believed (Ray, 2010). Intracellularly, accumulating evidence suggests a central role of TrkB/BDNF (brain-derived neurotrophic factor) and mTOR signaling pathways in the neuroplastic and behavioral effects of psychedelics (Ly et al., 2018, 2021; De Gregorio et al., 2021; Inserra et al., 2021a). Interestingly, although ketamine binds to a completely different receptor (NMDAR), its pro-synaptogenesis and antidepressant effects also engage BDNF and mTOR signaling (Li et al., 2010; Autry et al., 2011; Liu et al., 2012; Zanos and Gould, 2018; Aguilar-Valles et al., 2021). Such convergence in intracellular signaling not only provides a molecular mechanism for the shared cellular and behavioral phenotypes of ketamine and psychedelic treatment, but also hints at the molecular etiology of the psychiatric abnormalities they both ameliorate. At the same time, future studies on the distinct signaling pathways engaged by these drugs (Zanos et al., 2016; Marinova et al., 2017; Wu et al., 2021) will help resolve the difference in their duration of effect (Hibicke et al., 2020) and other pharmacological characteristics.

Many questions regarding the neuroplastic effects of psychedelics remain to be addressed. Are the dendritic spine dynamics localized to certain hotspots on the dendrite, specific to particular inputs, or uniformly distributed across the dendritic arbor? What are the functional implications of the observed structural plasticity? For example, are induced new synapses stronger and more active? Do different types of psychedelics, with their complex pharmacology, induce neuroplasticity with different spatiotemporal patterns in specific types of neurons? Moreover, it is important to clarify the molecular and circuit underpinnings of the differential effects generated by psychedelics and their analogs. Finally, how do the hallucinogenic and neuroplastic effects interact and contribute to the therapeutic potential of psychedelics? To address these questions, a cross-disciplinary collaboration is needed among chemists, molecular/cell biologists, synaptic physiologists, and clinical neuroscientists.

## Author Contributions

KL, JB, YZ, and JL: wrote the manuscript. All authors contributed to the article and approved the submitted version.

## Funding

This work was supported by grants from the National Institute of Mental Health (R01MH109475), the National Institute of Child Health and Human Development (R21HD101266), the National Institute on Aging (R01AG071787), and a Max Planck Fellowship at the Max Planck Florida Institute for Neuroscience to YZ.

## Conflict of Interest

The authors declare that the research was conducted in the absence of any commercial or financial relationships that could be construed as a potential conflict of interest.

## Publisher's Note

All claims expressed in this article are solely those of the authors and do not necessarily represent those of their affiliated organizations, or those of the publisher, the editors and the reviewers. Any product that may be evaluated in this article, or claim that may be made by its manufacturer, is not guaranteed or endorsed by the publisher.
